# The use of the computerized version of quality of life and health status questionnaires in a community sample in southern Brazil

**DOI:** 10.1590/S1516-31802011000100007

**Published:** 2011-01-06

**Authors:** Carlos Zubaran, Ivanor Tres

**Affiliations:** I MD, PhD. Conjoint associate professor, Department of Psychiatry, Blacktown Hospital, School of Medicine, University of Western Sydney, Australia.; II Researcher, School of Physical Education, Universidade de Caxias do Sul (UCS), Caxias do Sul, Rio Grande do Sul, Brazil.

**Keywords:** Quality of life, Health status, Medical informatics applications, Questionnaires, Brazil, Qualidade de vida, Nível de saúde, Aplicação de informática médica, Questionários, Brasil

## Abstract

**CONTEXT AND OBJECTIVE:**

Computerized data collection is an efficient process and well accepted by patients with different disorders. Although computer-based systems have been used to assess health status and quality of life in various areas of healthcare, there is a lack of studies to investigate the effectiveness of these instruments in Brazil. The aims here were to assess the usability of the Portuguese-language versions of the Personal Health Scale (PHS) and the Multicultural Quality of Life Index (MQLI) in southern Brazil and to determine the correlation between these two questionnaires.

**DESIGN AND SETTING:**

This was a cross-sectional community-based survey in which participants completed computerized versions of these two questionnaires.

**METHODS:**

In a survey conducted in 16 different locations, 458 volunteers completed both questionnaires. Pearson correlation coefficients were generated between the scores of the two questionnaires. The inclusion criteria allowed all volunteers who were able to understand the questions in both questionnaires to participate in the study.

**RESULTS:**

The percentage of proper data collection via the computerized versions of the two questionnaires combined was 97.45%. A significant correlation (P < 0.01) between the PHS and the MQLI was observed.

**CONCLUSION:**

The computerized versions of the PHS and MQLI demonstrated efficient data collection patterns during the field survey trials. Health-related issues were significantly correlated with the overall experience of wellbeing and quality of life. The computerized versions of the PHS and MQLI are valid tools for research and clinical use in Brazil.

## RESUMO

**CONTEXTO E OBJETIVO:**

A coleta computadorizada de dados é um processo eficiente e bem aceito por pacientes com diferentes enfermidades. Embora os sistemas computadorizados tenham sido usados para avaliar o estado de saúde e a qualidade de vida em várias áreas da assistência de saúde, há falta de estudos que investiguem a eficiência desses instrumentos no Brasil. O objetivo deste estudo foi avaliar a aplicabilidade da versão em Português da Escala de Saúde Pessoal (PHS) e do Índice Multicultural de Qualidade de Vida (MQLI) no Sul do Brasil, bem como determinar a correlação existente entre estes dois questionários.

**TIPO DE ESTUDO E LOCAL:**

Este é um estudo transversal, de base comunitária, no qual participantes completaram versões computadorizadas dos dois questionários.

**MÉTODOS:**

Em um estudo realizado em 16 locais diferentes, 458 voluntários preencheram os dois questionários. Foram gerados coeficientes de correlação de Pearson entre a pontuação de ambos questionários. Os critérios de inclusão permitiram que todos os voluntários capazes de entender as questões de ambos questionários participassem do estudo.

**RESULTADOS:**

O percentual de coleta de dados adequada através da versão computadorizada de ambos os questionários, combinados, foi de 97,45%. Foi observada uma correlação significativa (P < 0,01) entre o PHS e o MQLI.

**CONCLUSÃO:**

As versões computadorizadas do PHS e do MQLI demonstraram padrões eficientes de coleta de dados das pesquisas de campo. Questões relacionadas a saúde foram significativamente correlacionadas com a experiência global de bem-estar e qualidade de vida. As versões computadorizadas do PHS e do MQLI são instrumentos válidos para a pesquisa e uso clínico no Brasil.

## INTRODUCTION

The field of health status and assessment of quality of life measurements has been evolving as a formal discipline with structured theoretical foundations and specific methodology for more than 30 years.^1^ During the latter part of the 20^th^ century, patients' impressions about their own health and wellbeing became a salient topic of investigation in healthcare.^2^

Quality of life has been defined as the individual's perception of his position in life within the context of cultural and value systems, in relation to his objectives, beliefs and expectations.^3^ Measurement of quality of life provides a benchmark against which the impact of disease and different treatments at the personal level can be measured.^4^ On the other hand, health status questionnaires not only provide parameters for monitoring the impact of disease activity and the effect of a given therapeutic intervention, but also indicate the need for medical assistance and the degree of disability presented by a given patient. An improvement in health status and quality of life is an important primary outcome in determining the therapeutic benefit.^5^ Clinicians and policymakers now agree on the importance of measuring both general and health-related quality of life.^6^

Lately, on-site computerized tools^7^ and personal digital assistants (PDAs)^8^ have been introduced to the array of resources available for measuring quality of life and other health-related constructs. Electronic assessment of health status and quality of life provides new opportunities for regular patient monitoring and ready access to relevant information.^9^ Different electronic devices are now available for health screening and monitoring purposes, including web-based,^10^ fixed and interactive center-based computers^10^ or mobile handheld devices.^11^

Computerized data collection is well accepted by patients and time-efficient in clinical settings.^12^ Electronic collection of information facilitates data transfer^13^ and analysis.^14^ Compliance with assessments conducted via handheld computers can be superior to procedures using a “pen and paper” format.^15^ Some computer-based assessment systems facilitate data collection for individuals with mental disorders.^16^ Although PDAs have been used successfully to record health status and quality of life in various areas of healthcare,^15,17-20^ additional studies to investigate the effectiveness of these instruments in different patient populations and regions have been recommended.^21^

## OBJECTIVE

The main objective of this study was to conduct, for the first time in Brazil, a field survey using the electronic versions of two questionnaires for specifically evaluating quality of life and health status. Field trials on new investigational tools are an essential requirement for assessing their ease of use, acceptance by the target population, reliability, accuracy and effectiveness. The usability of the computerized versions of the Personal Health Scale (PHS) and the Multicultural Quality of Life Index (MQLI) were investigated in a community sample in southern Brazil. In this study, the authors also examined the correlation between these two questionnaires, as well as the amount of data loss during the surveys.

## MATERIAL AND METHODS

### Subjects

This study evaluated 470 adults in 16 localities close to the city of Caxias do Sul, which is one of the main urban hubs in Brazil's southernmost state of Rio Grande do Sul. These localities were included in a series of community programs that are developed annually by the Universidade de Caxias do Sul (UCS). As part of these community programs, academic staff and students from UCS visit a series of districts and cities on specific dates with the purpose of providing information and assistance within a myriad of fields ranging from healthcare to legal matters. These activities are advertised beforehand via a variety of media campaigns. In the present study, the data collection took place in all localities in conjunction with the three main institutional community programs sponsored by UCS, which are *Agita Caxias* (“Get Moving, Caxias”), Programa de Ação Comunitária (“Community Action Program”) and Projeto UCS Saúde e Cidadania (“UCS Project on Health and Active Citizenship”).

Potential participants were invited by word-of-mouth to respond to the electronic questionnaire. Participants were attending the community fair and their health statuses were unknown to the interviewer. The criteria for participating in this survey included the ability to understand both the aim of the study and the complete content of the questions in both questionnaires. The interviewers positioned themselves at the medical assessment and legal advice stands during the local community fairs. This study was endorsed by the institution's Research Ethics Committee. Before answering the questionnaires, the survey participants signed a brief consent form in which they declared that they voluntarily agreed with all the procedures implicated in this project.

All the volunteers were native Portuguese-speakers. Illiterate or semi-illiterate volunteers were also included in the research protocol, in a category defined as participants who were able to read but unable to write and/or who had limited knowledge or understanding, especially of technical subjects.

### Software development

A computer programmer from the UCS Data Processing Center was contracted to develop the software. The tool used for designing the electronic versions of the questionnaires was the eMbedded Visual Tools 3.0 - 2002 Free Edition for the Windows CE/Mobile platform. In terms of language features, Visual Basic was used. The local data storage was developed on a Pocket Access database manager. A Dell Axim X30 handheld computer was used during the entire study.

### Data administration

The study participants completed the questionnaires under minimal guidance from the trained examiners, who followed standardized instructional procedures. All the participants were initially instructed about how to complete the questionnaires. The instruction comments were standardized for all volunteers. The instructions were repeated until the volunteers confirmed that they had completely understood them. Occasionally, specific questions not considered in the initial instruction procedure were answered on a one-by-one basis. Special attention was given to illiterate or semi-illiterate volunteers in order to confirm that full understanding was attained.

The questions were read once, aloud and clearly, one-by-one for each interviewee. The handheld computer screen displaying the question under scrutiny was shown concomitantly to each volunteer. The interviewer entered the score chosen by the survey volunteer for each question. When questions were mistakenly skipped or data were lost during the interview process, the procedure was continued to completion, regardless, since the answers recorded in the handheld computer could not be erased or changed later.

After an entire session of data gathering had been completed in a given locality, the data stored in the handheld computer was transferred to a mainframe computer in the main research laboratory. The survey data were then converted to Microsoft Excel spreadsheets. Finally, visual accuracy checks were performed in order to confirm correct data recording. Satisfactory performance was defined as completion of all 20 questions included in both questionnaires. The accepted shortfall levels included instances of difficulty in understanding the questions of the questionnaires.

### Instruments

The MQLI is a visual analog scale composed of 10 dimensions, in which each dimension presents integer values from 1 to 10. This arrangement is applicable for both “pen and paper” and electronic scale formats. The dimensions include physical and emotional wellbeing, spiritual fulfillment, social functioning, community and services support as well as the overall perception of life. The magnitude of the score parallels the intensity or quality of the construct, in that higher scores indicate an elevated perception of quality of life.

The PHS is a 10-item instrument for self-rated assessment of general health status. It is an ordinal scale that measures the frequency of dimensions numerically from zero to three, based on dis tinct levels of presence and/or frequency of a given phenomenon. The PHS includes questions relating to areas such as sleep, mood, nervousness, fatigue and functional capacity. The score magnitude of each domain parallels the frequency of each correlated dysfunction or symptom, so that the higher the overall score on the instrument is, the lower the personal health status will be.

Versions of the MQLI and the PHS have already been validated in a range of languages.^3,22,23^ In developing both psychometric tools, particular attention was paid to multicultural issues.^24^ The Portuguese versions of the PHS and the MQLI were adapted to this language and tested in Brazil, and their respective psychometric properties are analyzed in the respective trial studies.^22,23^

## RESULTS

### Data collection

Out of the entire sample of 470 volunteers, two (one from each gender) were unable to respond to both questionnaires because of overt cognitive difficulties in understanding the questions. Information from three volunteers was not recorded in the PHS questionnaire due to human failure and electronic problems with the handheld computer. Three questions from the MQLI and five questions from the PHS were “skipped” by the handheld computer and lost during the interviews. The final subsamples of completers for the MQLI and the PHS were, respectively, 465 and 460 respondents. The final collective group of volunteers who responded to all questions from both questionnaires consisted of 458 volunteers: this figure was considered for correlational analyses only. Therefore, the combined completion rate for the computerized format of both questionnaires was 97.45%.

No refusal to complete either of the questionnaires was observed after the survey participants had signed the consent form. Although not formally recorded, it was noted that many of the volunteers had never answered a computerized questionnaire previously.

### Demographic data

The gender distribution among the whole sample of 470 volunteers was 262 women (55.7%) and 208 men (44.3%). In the subsample of complete MQLI respondents, 260 were women (55.9%) and 205 were men (44.1%), whereas in the subsample of complete PHS respondents, 256 were women (55.7%) and 204 were men (44.3%). In terms of age, the mean age for the entire sample of 470 volunteers was 37.68 years (standard deviation, SD = 13.07). In the subsample of complete MQLI respondents, the mean age was 37.63 years, whereas in the subsample of complete PHS respondents, the mean age was 37.59. In terms of region of origin, the total sample was divided according to 16 regional locations, including cities and districts, which are all listed in **Table 1**.

**Table 1.** Survey participants who answered all the questions of both questionnaires according to the different locations surveyed and the correlation of quality of life and health status scores

Locations	n	MQLI	PHS	*r*
		Mean Score (SD)	Mean Score (SD)	
Caxias do Sul	110	81.24 (10.12)	5.81 (3.58)	- 0.524*
São Marcos	30	81.5 (9.06)	7 (3.45)	- 0.434†
Criúva	12	84.58 (11.86)	4.58 (3.23)	- 0.494
Mulada	17	81.29 (13.37)	5 (3.7)	- 0.746*
Bom Princípio	29	79.52 (12.69)	6.66 (3.01)	- 0.667*
Monte Alegre dos Campos	30	83.3 (9.51)	6.47 (3.56)	-0.365†
Vacaria	34	78.24 (13.79)	8.35 (4.20)	-0.583*
Arroio do Ouro	13	84.08 (10.14)	5.92 (2.25)	- 0.562†
Vale Real	17	81.53 (8.25)	6.53 (4.26)	- 0.435
Vila Frangosul	18	77.67 (12.07)	6.78 (3.59)	- 0.503†
Veranópolis	38	81.29 (8.08)	6.89 (3.42)	- 0.406†
Nova Prata	30	77.8 (8.5)	7.03 (3.07)	- 0.468*
Nova Roma do Sul	19	78.32 (8.68)	6.63 (3.04)	- 0.496†
Muitos Capões	19	81.47 (10.47)	7.58 (3.04)	- 0.363
Vila Cristina	13	79.85 (9.56)	6.77(2.80)	- 0.542
Canela	29	83.72 (10.79)	5.48 (3.44)	- 0.334
**Total**	**458**	**80.82 (10.45)**	**6.46 (3.51)**	- 0.501*

* correlation significant at the 0.01 level;

† correlation is significant at the 0.05 level

MQLI = Multicultural Quality of Life Index; PHS = Personal Health Scale

### Score variance according to location

The statistical analyses were chosen based on a homogenous paradigm relating to the variance observed within these groups. One-way analysis of variance (Anova) was performed to evaluate the relationship between region of origin or locality and the total MQLI scores, which revealed the following values: *F* (15, 449) = 0.949, P = 0.509. Similarly, one-way Anova was conducted to evaluate the relationship between location of origin and the total PHS scores, which revealed a significant result: *F* (15, 444) = 1.821, P = 0.03. A post-hoc analysis was also conducted, using the Tukey HSD test for multiple comparisons. This latter analysis revealed a significant difference between the PHS scores of sub-samples from two cities: Caxias do Sul and Vacaria.

The mean MQLI scores and standard deviations of all the volunteers who also answered all the PHS questions are listed in **Table 1** according to locality.

### Pearson product-moment correlation coefficients

Correlation coefficients were computed among the overall scores (the sum of the scores for all ten questions) from the PHS and MQLI of all volunteers who answered all the questions from both questionnaires (n = 458). The statistical analysis yielded a Pearson correlation of - 0.501 with P < 0.01 (two-tailed). The correlation coefficients according to location of origin for all the volunteers who answered all the questions from both questionnaires are presented in **Table 1**.

## DISCUSSION

A significant correlation between the concepts of quality of life and health status, as evaluated by the Portuguese versions of the PHS and MQLI, was observed using the electronic versions of these questionnaires. This is consistent with the finding of a correlation between the scores of the PHS and MQLI using the pen-and-paper methodology during the trial studies on both questionnaires.^25^ This evidence contrasts with the findings from one of the few studies designed to investigate a possible correlation between health status and quality of life, which revealed no overlapping performances between a disease-specific quality of life questionnaire and a generic health assessment tool (short form, SF-36) for assessing volunteers affected by allergic conditions.^26^

The current study presents limitations that hinder subsequent interpretations regarding the possible role played by socioeconomic factors in explaining both quality of life and health status, since socioeconomic status was not evaluated in this study. Although it is possible to investigate socioeconomic status using specific questionnaires, the aim in the present study was to minimize interviewing time as much as possible, so that volunteers could easily tolerate the interview process. Furthermore, this study did not compare the electronic versions of the questionnaires with their respective “pen and paper” counterparts. The fact that the correlation between the two electronic questionnaires replicates the previously reported correlation between the two “pen and paper” versions of the same instruments, as mentioned above, suggests that consistency has been maintained in the computerized versions of the questionnaires.

The strengths of this study include the negligible gender disparity in the sample, along with the size and geographical distribution of the sample, which encompassed several localities in southern Brazil. These study characteristics provide a considerable demographic representation of a wider sample size. Nonetheless, epidemiological investigations on both quality of life and health status in southern Brazil are beyond the scope of this study. Such a valid and desirable research undertaking would require an even larger sample size, with analyses of different sub-groups as well as a wider distribution of locations.

During the field trials on the electronic format of the two questionnaires, limited loss of data was observed, and this was within the accepted shortfall levels observed in other trials on electronic assessment tools.^12^ Electronic data transfer was an advantageous method for data analysis, since data transmitted electronically can be incorporated directly into a database. Furthermore, the data was easily stored as spread-sheet documents within a commonly used type of software (Microsoft Excel). In fact, the importance of a well-designed computer interface has been highlighted as a crucial factor for successful implementation of computerized patient questionnaires.^27^

These computerized questionnaires were easy to use and well accepted, since no refusals to complete the interview were observed after participants had signed the consent form. The data-gathering process was conducted mostly by a single interviewer and data transfer to a desktop environment was also very efficient. An earlier, similar study demonstrated that, irrespective of previous computer use, most participants reported that the electronic version of the quality of life questionnaire was easy to use and understand as well as a quick-to-use tool.^19^ Computerized questionnaires would facilitate data gathering, recording and transfer to a stable databank environment, which consequently minimizes error rates and improves productivity. Future studies should also investigate interviewees' subjective impressions of the use of computerized health-related questionnaires according to different categories such as age, gender, educational background and socioeconomic status.

## CONCLUSION

The current study demonstrates that the computerized formats of the Portuguese versions of the Multicultural Quality of Life Index and the Personal Health Scale are effective electronic tools for health status monitoring purposes and epidemiological research in Brazil. The computerized questionnaires were tested on a community-derived sample of individuals and achieved satisfactory performance. Computerized quality of life and health status questionnaires can be effectively used at a community level and with large samples. Additional studies that include rural areas may be worthwhile to generate a more representative picture of both regional and nationwide health status and quality of life standards.

## References

[1] Assessing health status and quality-of-life instruments: attributes and review criteria. Qual Life Res. 2002;11(3):193-205. 12074258 10.1023/a:1015291021312

[2] von Engelhardt D. Patient vs. disease in medicine: Historical perspectives and contemporary concerns. J Nephrol. 2004;17(4):611-8. 15372428

[3] Mezzich JE Ruipérez MA Pérez C. The Spanish version of the quality of life index: presentation and validation. J Nerv Ment Dis. 2000;188(5):301-5. 10830568 10.1097/00005053-200005000-00008

[4] Thompson DR Roebuck A. The measurement of health-related quality of life in patients with coronary heart disease. J Cardiovasc Nurs. 2001;16(1):28-33 11587238 10.1097/00005082-200110000-00005

[5] Treasure T. The measurement of health related quality of life. Heart. 1999;81(4):331-2 10092552 10.1136/hrt.81.4.331 PMC1728985

[6] Guyatt GH. A taxonomy of health status instruments. J Rheumatol. 1995;22(6):1188-90. 7674253

[7] Le PP Kohane IS Weeks JC. Using a pen-based computer to collect health-related quality of life and utilities information. Proc Annu Symp Comput Appl Med Care. 1995:839-43. 8563410 PMC2579212

[8] Kvien TK Mowinckel P Heiberg T. Performance of health status measures with a pen based personal digital assistant. Ann Rheum Dis. 2005;64(10):1480-4. 15843456 10.1136/ard.2004.030437 PMC1755226

[9] Kvien TK Uhlig T. Quality of life in rheumatoid arthritis. Scand J Rheumatol. 2005;34(5):333-41. 16234180 10.1080/03009740500327727

[10] Bush N Donaldson G Moinpour C. Development, feasibility and compliance of a web-based system for very frequent QOL and symptom home self-assessment after hematopoietic stem cell transplantation. Qual Life Res. 2005;14(1):77-93. 15789943 10.1007/s11136-004-2394-2

[11] Gaertner J Elsner F Pollmann-Dahmen K Radbruch L Sabatowski R. Electronic pain diary: a randomized crossover study. J Pain Symptom Manage. 2004;28(3):259-67. 15336338 10.1016/j.jpainsymman.2003.12.017

[12] Wilson AS Kitas GD Carruthers DM. Computerized information-gathering in specialist rheumatology clinics: an initial evaluation of an electronic version of the Short Form 36. Rheumatology (Oxford). 2002;41(3):268-73. 11934962 10.1093/rheumatology/41.3.268

[13] Drummond HE Ghosh S Ferguson A Brackenridge D Tiplady B. Electronic quality of life questionnaires: a comparison of pen-based electronic questionnaires with conventional paper in a gastrointestinal study. Qual Life Res. 1995;4(1):21-6. 7711686 10.1007/BF00434379

[14] Lane SJ Heddle NM Arnold E Walker I. A review of randomized controlled trials comparing the effectiveness of hand held computers with paper methods for data collection. BMC Med Inform Decis Mak. 2006;6:23. 16737535 10.1186/1472-6947-6-23 PMC1513201

[15] Walker I Sigouin C Sek J. Comparing hand-held computers and paper diaries for haemophilia home therapy: a randomized trial. Haemophilia. 2004;10(6):698-704. 15569164 10.1111/j.1365-2516.2004.01046.x

[16] Vieweg BW DiFranco B. The use of automated assessment with seriously mentally ill clients. Behav Healthc Tomorrow. 1995;4(1):37-41. 10140329

[17] VanDenKerkhof EG Goldstein DH Blaine WC Rimmer MJ. A comparison of paper with electronic patient-completed questionnaires in a preoperative clinic. Anesth Analg. 2005;101(4):1075-80. 16192524 10.1213/01.ane.0000168449.32159.7b

[18] DeVore PA. A computerized geriatric assessment designed for use in primary care physicians' offices. Md Med J. 1994;43(3):257-64. 8201854

[19] Carlson LE Speca M Hagen N Taenzer P. Computerized quality-of-life screening in a cancer pain clinic. J Palliat Care. 2001;17(1):46-52. 11324185

[20] Taenzer PA Speca M Atkinson MJ. Computerized quality-of-life screening in an oncology clinic. Cancer Pract. 1997;5(3):168-75. 9171553

[21] Taenzer P Bultz BD Carlson LE. Impact of computerized quality of life screening on physician behaviour and patient satisfaction in lung cancer outpatients. Psychooncology. 2000;9(3):203-13. 10871716 10.1002/1099-1611(200005/06)9:3<203::aid-pon453>3.0.co;2-y

[22] Zubaran C Persch K Tarso D Ioppi AE Mezzich J. The Portuguese version of the personal health scale: a validation study in southern Brazil. Clinics (Sao Paulo). 2007;62(4):419-26. 17823704 10.1590/s1807-59322007000400008

[23] Zubaran C Persch KN Tarso D Ioppi AEE Mezzich JE. Estudo inicial para o desenvolvimento da versão em português do índice multicultural de qualidade de vida [Initial study for the development of the portuguese version of the multicultural quality of life index]. Arquivos Brasileiros de Psiquiatria, Neurologia e Medicina Legal. 2004;98(3). Available from: http://www.aperjrio.org.br/publicacoes/revista/2004/estudo.asp. Accessed in 2010 (Nov 4).

[24] Guillemin F Bombardier C Beaton D. Cross-cultural adaptation of health-related quality of life measures: literature review and proposed guidelines. J Clin Epidemiol. 1993;46(12):1417-32. 8263569 10.1016/0895-4356(93)90142-n

[25] Zubaran C Persch K Tarso D Ioppi AE Mezzich J. The correlation between health status and quality of life in southern Brazil [A correlação entre estado de saúde e qualidade de vida no sul do Brasil]. Sao Paulo Med J. 2008;126(5):257-61. 19099158 10.1590/S1516-31802008000500003 PMC11026044

[26] Terreehorst I Duivenvoorden HJ Tempels-Pavlica Z. Comparison of a generic and a rhinitis-specific quality-of-life (QOL) instrument in patients with house dust mite allergy: relationship between the SF-36 and Rhinitis QOL Questionnaire. Clin Exp Allergy. 2004;34(11):1673-7. 15544589 10.1111/j.1365-2222.2004.02096.x

[27] Lutner RE Roizen MF Stocking CB. The automated interview versus the personal interview. Do patient responses to preoperative health questions differ? Anesthesiology. 1991;75(3):394-400. 1888045

